# Compression of the Superior Vena Cava by an Interatrial Septal Lipoma: A Case Report

**DOI:** 10.1155/2013/945726

**Published:** 2013-08-01

**Authors:** R. Grech, A. Mizzi, S. Grech

**Affiliations:** ^1^Department of Radiology, Beaumont Hospital, Dublin 9, Ireland; ^2^Department of Medical Imaging, Mater Dei Hospital, B'Kara MSD 2090, Malta; ^3^Department of Orthopaedics, Mater Dei Hospital, B'Kara MSD 2090, Malta

## Abstract

Primary cardiac tumours are rare; their prevalence ranges from 0.0017% to 0.28% in various autopsy series. Cardiac lipomas are well-encapsulated benign tumours typically composed of mature fat cells, and their reported size ranges from 1 to 15 cm. They are usually seen in the left ventricle and the right atrium. Lipomas are true neoplasms, as opposed to lipomatous hypertrophy of the interatrial septum, which is a nonencapsulated hyperplastic accumulation of mature and foetal adipose tissue. Cardiac lipomas occur in patients of all ages, and the frequency of occurrence has been found to be equal in both sexes. Patients are usually asymptomatic, although the manifestation of symptoms depends upon both size and location of the tumour. We present the case of a patient with an interatrial septal lipoma, causing obstruction of the superior vena cava.

## 1. Case Report

A 45-year-old woman was referred by her general practitioner, with a history of dyspnoea and occasional cyanotic spells. She described a three-month history of progressive shortness of breath, tachypnoea, and headaches. Physical examination revealed facial oedema and cyanosis of the head and shoulders. The superficial veins of the upper extremities and neck were dilated. She had a normal pulse rate and a regular rhythm. Her blood pressure was recorded at 115/70. No heart murmur or thrill was present. There was no hepatomegaly or lower-extremity oedema.

**Figure 1:**
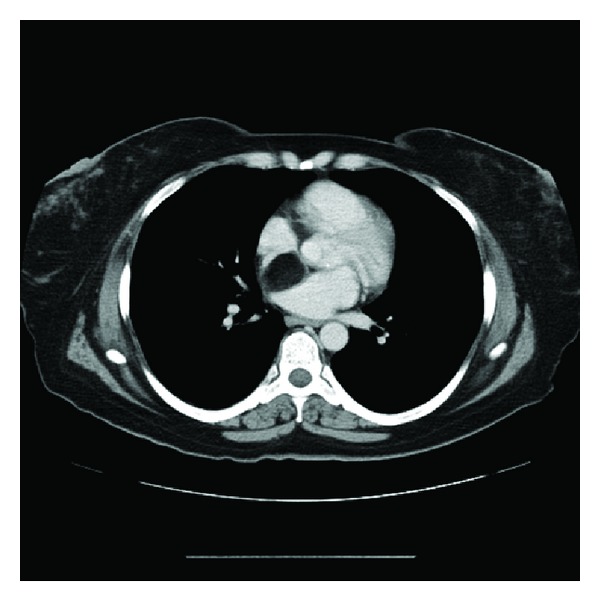
Axial CT image with intravenous contrast, showing the interatrial septal lipoma (encapsulated).

**Figure 2:**
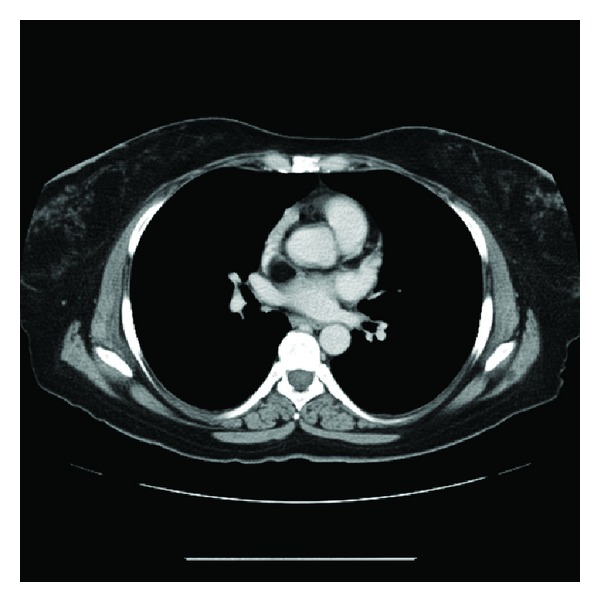
A second axial image, more cephalad, shows the septal lipoma, compressing the superior vena cava.

**Figure 3:**
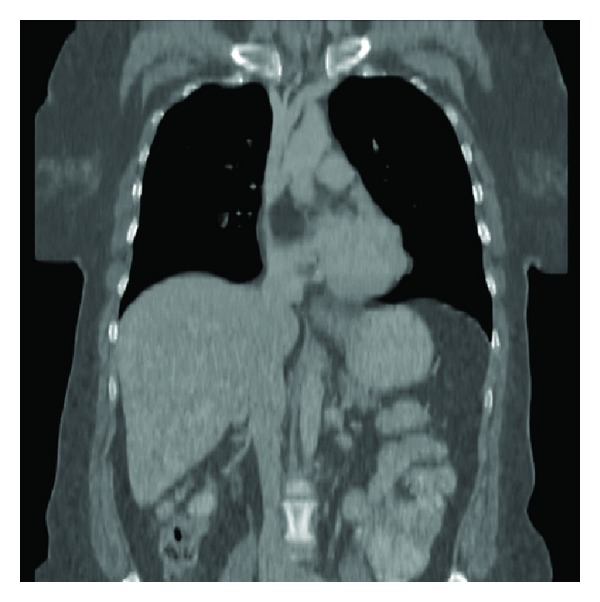
Coronal reconstruction, showing the entire length of the superior vena cava, that is, from the right and left brachiocephalic veins down to the right atrium. Compression by the septal lipoma is seen, at the point where the SVC enters the atrium.

**Figure 4:**
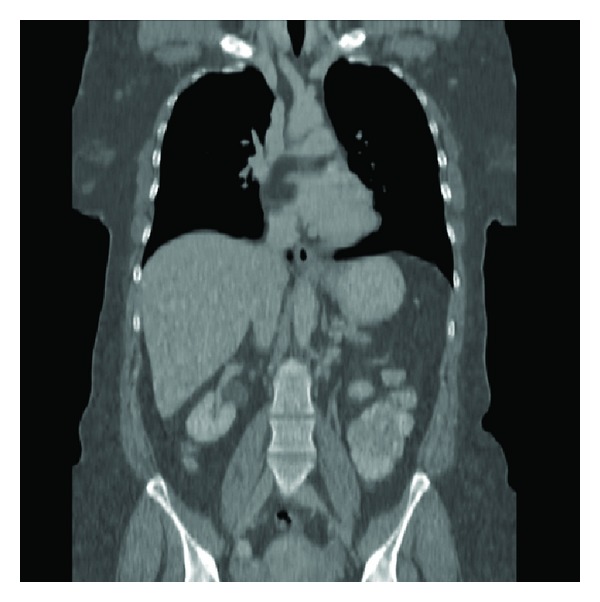
Coronal CT reconstruction shows how the superior vena cava tapers distally, as it is compressed by the lipoma.

The electrocardiogram was negative for ischemia and showed sinus rhythm. Laboratory investigations were normal, except for a mildly lowered haemoglobin level. A radiograph of the chest did not reveal pulmonary vascular congestion, pulmonary oedema, or cardiomegaly. Subsequent computed tomography (CT) of the chest disclosed a low-density soft-tissue mass in the upper part of the interatrial septum that was compressing the superior vena cava (SVC). The mass measured 3 × 5 cm and had a Hounsfield number of −100, consistent with fat (Figure 1). The lumen of the SVC was significantly narrowed (Figures 2, 3, and 4), as shown on the CT scan by the anterior displacement of contrast material caused by extraluminal compression. There were no other significant positive findings.

The patient categorically refused any operative intervention as was advised by the cardiothoracic surgeons. She decided to decline offers for further investigations including echocardiography.

## 2. Discussion

Primary tumours of the heart are far less common than metastatic tumours. The incidence of metastatic tumours to the heart is 20–30 times that of primary lesions. About 75% of all primary cardiac tumours are benign. Most malignancies are various forms of sarcoma.  Table 1 shows a classification of primary cardiac tumours with their relative frequency [1]. The majority of patients are asymptomatic, although the manifestation of symptoms depends upon the size and location of the tumour.

The symptoms of benign tumours are often ambiguous and have an intriguing capacity to simulate more common forms of heart disease, for example, heart failure, rhythm and conduction defects, syncopal attacks, and embolism [2]. Metastatic neoplasms involving the heart may present clinical characteristics identical to those demonstrated by primary tumours. Benign nonmyxomatous neoplasms of the heart are rare, and lipomas are among those least often encountered.

Cardiac lipomas were first described by Albers [3] in 1856, and approximately 60 cases have since been reported [4]. Lipomas are tumours of mesodermal origin that infrequently arise from the heart. Histologically these tumours resemble lipomas situated elsewhere in the body. About 50% arise subendocardially, 25% subepicardially, and 25% from the myocardium [1]. Although lipomas have a relatively soft tissue consistency, cases have been reported in which lipomas were found to compress vascular structures, but our search of the English literature failed to identify any reports of interatrial septal lipomas that compressed the SVC. We did however find a case where a mediastinal lipoma caused SVC compression [5]. 

Several diagnostic modalities [6] are available, and these are usually specific for demonstrating a cardiac mass lesion. Plain chest radiography may show secondary effects caused by the tumour and in some instances may also directly reveal the tumour when calcification is present. Myxomas are the most common tumours showing this calcification. Similarly, an ECG gives only indirect evidence by showing chamber enlargement or rhythm disturbances. Angiography and especially cardiac catheterization may be hazardous in such patients since they may cause tumour embolisation. Apart from this risk several conditions may give false positives, and these include hematoma, abscess, thrombus, and aortic aneurysm. 

Echocardiography is one of the most important modalities available today for imaging cardiac tumours. It has the advantage of being a noninvasive technique. Two-dimensional echocardiography has many advantages over standard M-mode studies and can easily determine the tumour size, shape, and mobility. Transoesophageal echocardiography provides an excellent view of both the atria and the atrial septum and can be used to detect some masses not revealed by transthoracic echocardiography. 

Computed tomography and magnetic resonance imaging are very helpful in that they not only demonstrate the lesion itself but also help to determine the degree of myocardial invasion as well as the involvement of pericardial and extra-cardiac structures. In fact these two modalities have become the gold standard examinations for the imaging of cardiac tumours in most centres.

All cardiac tumours are potentially lethal because of the risks of valvular obstruction, embolism, and arrhythmias [7]. Lipomas arising from the free left ventricular wall can be easily excised, but intramural atrial lesions require the use of cardiopulmonary bypass for resection. Prompt surgical excision of cardiac tumours often results in a complete cure, affording these patients an excellent long-term prognosis.

## Figures and Tables

**Table 1:** Relative frequency of primary cardiac tumours [1].

Benign (75%)		Malignant (25%)	
Myxoma	30%	Angiosarcoma	8%
Lipoma	10%	Rhabdomyosarcoma	5%
Papillary fibroelastoma	8%	Fibrosarcoma	3%
Rhabdomyoma	6%	Mesothelioma	3%
Fibroma	3%	Lymphoma	2%
Haemangioma	2%	Leiomyosarcoma	1%
Teratoma	1%		

## Abbreviations

ECG: Electrocardiogram
SVC: Superior vena cava.
